# The functional imperative in high-grade glioma

**DOI:** 10.1038/s12276-025-01614-x

**Published:** 2026-01-08

**Authors:** Laura Shih Hui Goh, Dexter Kai Hao Thng, Yvonne Li En Ang, Dean Ho, Tan Boon Toh, Andrea Li Ann Wong

**Affiliations:** 1 Yong Loo Lin School of Medicine, National University of Singapore, Singapore, Singapore; 2 The N.1 Institute for Health, National University of Singapore, Singapore, Singapore; 3 The Institute for Digital Medicine (WisDM), Yong Loo Lin School of Medicine, National University of Singapore, Singapore, Singapore; 4 Department of Hematology-Oncology, National University Cancer Institute, Singapore, Singapore; 5 NUS Centre for Cancer Research, Yong Loo Lin School of Medicine, National University of Singapore, Singapore, Singapore

**Keywords:** CNS cancer, Cancer therapy, Translational research

## Abstract

Precision oncology has emerged as a promising strategy for treating high-grade gliomas, yet its clinical impact has been disappointing, with over 300 clinical trials on targeted therapies failing to yield substantial improvements in patient outcomes. Current approaches primarily focus on static, marker-driven tumor features, which capture only a small portion of the complex biology that governs therapeutic responses. Functional precision oncology (FPO) offers a complementary approach, enhancing treatment selection in a personalized manner by dynamically testing patient-derived tumor cells against a range of available therapeutic agents. Here this review examines both historical and contemporary treatment strategies for high-grade gliomas and explores underlying reasons for the limited success of multiple precision oncology initiatives. We demonstrate how the incorporation of FPO in the armamentarium of glioma therapies may address these challenges and outline its proposed role as well as the practical considerations in utilizing FPO for clinical decision-making in patients with glioma.

## Dynamic testing transforms precision oncology for gliomas

Precision oncology aims to tailor cancer treatments based on individual genetic profiles. This study highlights the challenges faced in using genomic data alone to predict treatment outcomes for high-grade gliomas. The researchers discuss the emergence of functional precision oncology (FPO) as a promising strategy. FPO involves testing a patient’s tumor cells directly against various drugs to identify effective treatments. This method considers the complex biology of tumors, which genomic data alone may not capture. In their study, the authors addresses the use of patient-derived models, such as organoids (3D cell cultures), to test drug responses. These models help identify personalized treatment options by simulating how tumors react to different therapies. The study concludes that FPO can complement genomic approaches, offering a more comprehensive understanding of tumor behavior and improving treatment strategies for high-grade gliomas.

This summary was initially drafted using artificial intelligence, then revised and fact-checked by the author.

## Introduction

The success of targeted therapies ushered in the era of precision oncology, a field focused on identifying and targeting cancer-specific alterations to guide more effective treatments. Pioneering therapies such as imatinib and trastuzumab, which revolutionized the management of chronic myeloid leukemia and HER2-positive breast cancer, respectively, laid the foundation for targeted approaches. Since then, the field has expanded substantially, fueled by large-scale cancer genomic projects aimed at uncovering actionable targets for therapeutic intervention^1^.

While numerous success stories illustrate the transformative potential of precision oncology, it is key to realize that these successes are exceptions rather than the rule. The effectiveness of targeted therapies often varies markedly depending on the cancer type and its underlying biological context. Glioblastoma (GBM), the most aggressive form of high-grade glioma (HGG), was the first cancer to be molecularly characterized by The Cancer Genome Atlas (TCGA)^2^. This landmark effort has since transformed glioma classification, culminating in the World Health Organization (WHO) 2021 classification, which integrates molecular diagnostics into the taxonomy of central nervous system (CNS) tumors^3^. Over the past two decades, more than 300 clinical trials exploring targeted therapies and 50 trials investigating immunotherapy have failed to show significant improvements in patient outcomes^4,5^. Efforts have focused on targeting genetic alterations implicated in HGG pathogenesis, including *EGFR* overexpression (evaluated through receptor tyrosine kinase inhibitors and ADC against EGFRvIII), *PTEN* loss (addressed by PI3K pathway inhibitors) and tumor angiogenesis (targeted by antiangiogenic agents such as bevacizumab). While a new wave of immuno-oncology studies is emerging, past immuno-oncology strategies, both as monotherapies and in combination therapies, have failed to establish a new standard of care (SOC)^5^. As a result, HGG continues to rank among the malignancies with the highest failure rates in cancer treatment.

Despite extensive molecular characterization, it is increasingly evident that measurable genomic data represents only a fraction of the intricate biology driving therapeutic responses in HGG. This highlights the need to better understand the complexity of HGG, in particular the dynamic interplay of factors such as cellular state plasticity, microenvironmental interactions and epigenetic modifications that collectively shape tumor behavior^6^. Crucially, an incomplete understanding of all the factors influencing therapeutic outcomes lends support to the inadequacy of current genomic-centric precision oncology approaches and clinical trials over the past two decades^7^.

More recently, functional precision oncology (FPO) has emerged as a complementary strategy that bridges the gap between static genomic data and dynamic tumor behavior by directly testing a patient’s tumor cells against therapeutic agents. This real-time assessment of drug efficacy provides a more objective basis for identifying effective treatments beyond genetic probabilities, and is especially valuable in cases such as HGG, where mutation-based approaches have failed to reliably predict clinical outcomes. By expanding the focus beyond targetable mutations alone, FPO enhances current precision oncology strategies, offering a more personalized and comprehensive evaluation of drug responses^8^. In practice, FPO workflows begin with the collection of patient-derived specimens to generate ex vivo models, such as 2D cells, 3D organoids or tissue explants, which are rapidly screened against a panel of drugs to quantify tumor cell responses using functional assays that measure viability, growth inhibition or apoptosis. The findings are subsequently integrated with molecular and clinical data, often through multidisciplinary tumor boards, to inform individualized treatment recommendations within clinically relevant timeframes. Studies in hematological malignancies and solid tumors, including HGG, have demonstrated the feasibility and predictive power of FPO, with clinical correlations and successful therapeutic outcomes^8–11^.

In this review, we provide a comprehensive overview of existing approaches in managing HGGs, outline three key pillars fundamental to precision oncology strategies and explore the unique biological context of HGG that may have limited the success of previous trials. Finally, we discuss how FPO offers a promising and innovative approach to address the shortcomings of genomics-based approaches, paving the way for more effective and personalized treatment strategies.

## The current treatment paradigm for HGGs: past trials, future challenges

Historically, HGG referred to a heterogeneous group of gliomas defined based on histopathological features supported by ancillary tissue-based tests (for example, immunohistochemistry), encompassing grade 3 and 4 tumors. More recently, the WHO 2021 classification advanced the role of molecular diagnostics in CNS tumor classification, dividing adult HGG into four subtypes: grade 3 oligodendroglioma (1p/19 co-deleted, *IDH*-mutant), grade 3 *IDH*-mutant astrocytoma, grade 4 *IDH*-mutant astrocytoma and grade 4 *IDH* wild-type GBM^3^. HGG carries a median overall survival (OS) of 14 months and a median progression-free survival (PFS) of 7 months, with tumor recurrence observed in over 90% of patients^8,9^. Unfortunately, no established SOC treatment exists at recurrence, for which patients have a median OS of 6.2 months^10^.

### Timeline of FDA-approved systemic therapies in HGG

**Fig. 1:**
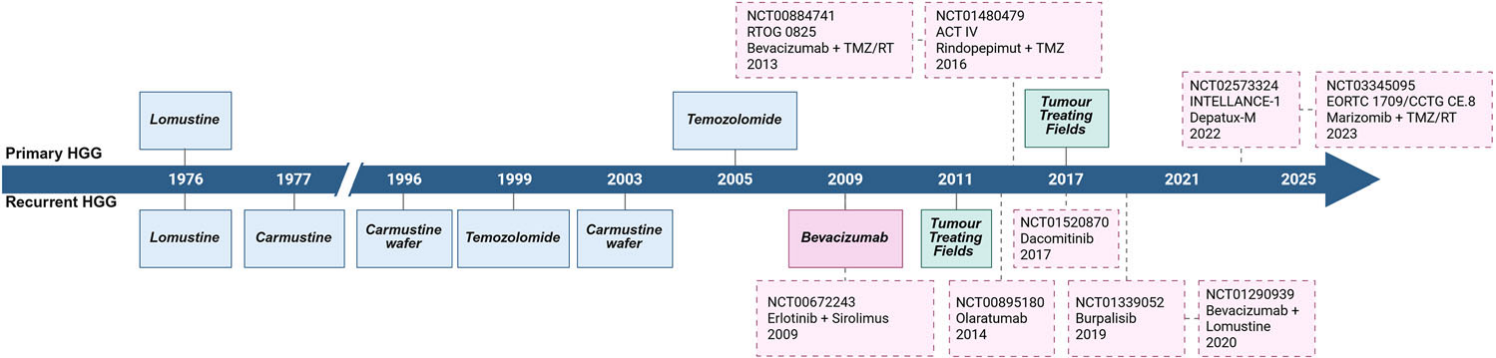
Timeline of FDA-approved modalities in HGG. A timeline illustrating the evolution of FDA-approved cancer therapies for HGG, categorized by their application in primary or recurrent disease. The modalities are color coded to illustrate their evolution, transitioning from chemotherapy (blue) to targeted therapies (pink) and device-based treatments (green). Key clinical trials that occurred during the corresponding periods are also shown.

There have been very few FDA-approved therapies for HGG (Fig. 1). Most approved therapies are traditional cytotoxic therapies belonging to the DNA alkylating class, including temozolomide (TMZ), lomustine and carmustine (both intravenous and wafer implants). Exceptions to this include bevacizumab and tumor-treating field (TTF) devices. In this review, we focus on treatment regimens used for high-grade astrocytic gliomas, which constitute the majority of HGGs in adults.

Maximal safe resection followed by radiotherapy remains the cornerstone of treatment for HGG, with its role established through studies dating back to the 1970s^11^. Before the introduction of TMZ, systemic therapy was predominantly based on cytotoxic agents such as lomustine and carmustine. These agents continue to be widely used for the management of recurrent HGG, although a universally accepted SOC treatment remains elusive^12^. The efficacy of lomustine was first demonstrated as part of the regimen comprising of procarbazine, lomustine, and vincristine (PCV), during which it was combined with procarbazine and vincristine following radiotherapy in newly diagnosed HGG^13^. Lomustine received FDA approval in 1976 for the treatment of primary and recurrent HGG; while its efficacy remains largely limited to tumors with *O*_6_-methylguanine DNA methyltransferase (MGMT) promoter methylation, it continues to be used in the recurrent setting regardless of MGMT status^14^. Similarly, carmustine demonstrated a survival benefit in two pivotal phase III trials, which showed that the addition of carmustine to surgery and radiotherapy improved survival, with a greater proportion of patients surviving beyond 18 months. Carmustine was subsequently approved by the FDA in 1977 for the treatment of recurrent HGG^15,16^.

The landscape of HGG management shifted notably in 2005, when Stupp et al. demonstrated superior survival outcomes with the addition of TMZ to radiotherapy in the first-line treatment of HGG^9^. This survival benefit was considered a therapeutic milestone for systemic therapy in HGG management and established the Stupp protocol as the new SOC. Since then, the evolving molecular understanding of HGG has led to countless trials evaluating chemotherapy, targeted therapy and immunotherapy, but none has succeeded in replacing the Stupp protocol.

Bevacizumab is a monoclonal antibody targeting vascular endothelial growth factor, inhibiting angiogenesis thereby restricting tumor vascularization. Several phase II and III trials have explored the inclusion of bevacizumab to chemoradiation with TMZ in the front-line treatment of HGG, including the phase III RTOG 0825 (NCT00884741) and phase III AVAGlio (NCT00943826). Despite showing a modest prolongation in PFS, these trials did not demonstrate a significant improvement in OS^17^. In 2006, a phase II study (NCT00345163) assessed the efficacy of bevacizumab, both as a monotherapy and in combination with irinotecan, for patients with recurrent HGG. The promising results from this study led to FDA approval of bevacizumab in 2009 as salvage therapy for recurrent HGG^18^. Subsequently, several other trials explored the use of bevacizumab in combination with various chemotherapy agents^17^. While the combination of lomustine and bevacizumab appeared promising in the phase II BELOB trial (NCT01290939) and the phase III EORTC 26101 trial (NCT01290939), these trials failed to meet their primary endpoint of an OS benefit^19^. Nonetheless, bevacizumab and lomustine remain the most commonly used systemic treatments for recurrent HGG so far^19^.

In recent years, TTFs, a noninvasive anti-cancer modality utilizing low-intensity, intermediate-frequency alternating electric fields, has become more widely accessible. The EF-11 trial (NCT00379470) in 2006 demonstrated the efficacy of TTFs in patients with recurrent HGG, leading to FDA approval in 2011 for its use in recurrent tumors. Subsequently, the EF-14 trial (NCT00916409) in 2009 showed that combining TTFs with maintenance therapy significantly improved OS in newly diagnosed patients, resulting in FDA approval in 2017. This pivotal study marked the first advancement in over a decade to extend OS for newly diagnosed HGG patients since the addition of TMZ to radiotherapy^20^. However, real-world uptake remains limited, with only 18–20% of eligible patients receiving TTF in recent multicenter cohorts, with optimal usage (≥75% daily, ≥18 h/day) achieved by only a minority^21,22^. Barriers include device burden, cost and quality-of-life considerations, contributing to variable adoption despite guideline endorsement^21,23^.

### Emerging therapeutic modalities in HGG

In recent years, emerging targeted therapies and immunotherapy approaches, supported by robust preclinical data, have been extensively investigated in HGG, as reviewed previously^4^. Despite substantial monetary investments and their representation in a large proportion of clinical trials, these therapies have largely failed to demonstrate meaningful improvements in survival outcomes^4,5^. Among the numerous strategies evaluated, bevacizumab remains the only FDA-approved targeted therapy for HGG. Here, we discuss the development of a few key therapies.

#### Targeted therapy

*EGFR* amplification is observed in approximately 50% of HGGs, with the EGFRvIII variant the most common alteration. So far, approximately 40 ongoing or completed trials have explored various strategies targeting EGFR, including small-molecule tyrosine kinase inhibitors, monoclonal antibodies, antibody–drug conjugates (ADC) and vaccines. However, despite the perceived importance of EGFR as a target in other cancers, such as non-small cell lung cancer and colorectal cancer, EGFR-targeted trials have largely failed to provide clinical benefit in HGG^24^. Several phase II trials investigating gefitinib and erlotinib showed no clinical efficacy in patients with newly diagnosed HGG, regardless of EGFR amplification or mutation status, attributable to the limited brain penetrance of these compounds^24–28^. While limited brain penetrance may contribute, emerging evidence suggests that context-specific resistance mechanisms may play a larger role. Interestingly, newer brain-penetrant EGFR inhibitors such as osimertinib, while effective in EGFR-mutant non-small cell lung cancer brain metastases^29,30^, have not demonstrated meaningful benefit in HGG, with reports showing rapid progression on the drug^31–33^. More recently, the fourth-generation EGFR inhibitor, silevertinib, is being investigated in an ongoing phase I/II trial (NCT05256290) for patients with recurrent GBM harboring EGFR alterations, with results currently pending^34^. Other strategies evaluating monoclonal antibodies, as well as the ADC, depatuxizumab mafodotin, have similarly yielded disappointing results, showing no improvement in clinical outcomes^35^. Several factors may underlie this unexpected discrepancy between preclinical promise and clinical failure, underscoring the unique challenges of targeting EGFR in HGGs.

Recent studies have demonstrated that while *EGFRvIII* is present in primary tumors, it is often undetectable in nearly half of recurrent tumors following SOC treatment. The loss of *EGFRvIII* expression in recurrent HGG is hypothesized to be driven by clonal selection and tumoral heterogeneity, where treatment pressures favor tumor subpopulations without *EGFRvIII* expression^36^. This suggests that tumor recurrence may not be dependent on *EGFRvIII*, raising important questions about its role in glioma biology. While *EGFRvIII* may be a critical driver in primary tumor initiation and early progression, it may no longer play a central role in the mechanisms driving treatment failure and tumor recurrence^36,37^ However, despite *EGFRvIII* loss, *EGFR* expression is rarely lost at recurrence, suggesting alternative resistance mechanisms, with the transition of EGFR from chromosomal to extrachromosomal DNA playing a potential role^38^. In addition, as gliomas are rarely dependent on a single gene or pathway, *EGFR* and *EGFRvIII* are likely to represent just one component of a broader, highly interconnected signaling network^39^. This complexity explains the limited clinical efficacy of therapies targeting individual molecular alterations, as such approaches fail to address the adaptive and multifaceted nature of HGG biology^4^.

The PI3K/AKT/mTOR pathway, a hallmark of HGG associated with poorer outcomes, has been the focus of numerous strategies supported by strong preclinical evidence^40^. Initial optimism arose from phase III trials showing that the mTOR inhibitor everolimus was able to reduce the volume of subependymal giant cell astrocytomas^41^. However, mTOR inhibitors in HGG have largely failed to demonstrate meaningful benefit^42^. These failures may stem from compensatory AKT activation via unregulated mTORC2 signaling and the absence of reliable predictive biomarkers to identify treatment-responsive patients. In the recently completed phase II/III GBM AGILE trial (NCT03970447), paxalisib, a potent oral selective brain-penetrant small-molecule PI3K/mTOR inhibitor, demonstrated a promising 3.8-month OS improvement compared to SOC treatment in the MGMT unmethylated subpopulation of newly diagnosed GBM, suggesting potential benefit in selected patients^42^. This has led to the design of an anticipated phase III first-line study of paxalisib, in addition to the ongoing studies of the drug in recurrent gliomas (NCT05009992 and NCT05183204)^43,44^.

*BRAF*^*V600E*^ mutations, which are present in 1–8% of HGGs, and up to 20% of cases in patients under 30 years of age, have emerged as potentially actionable mutation in HGG^45^. Several case reports have highlighted rapid and occasionally complete responses to BRAF inhibitors (BRAFi) and BRAF–MEK inhibitor combinations, a rarity seen in the setting of HGGs^46^. This has driven ongoing research, with larger basket trials reporting similar encouraging outcomes. Notably, the phase II ROAR trial (NCT02034110) demonstrated a 33% objective response rate in 31 patients with HGG, including three complete responses^47^. While further studies are needed to understand the long-term clinical course of BRAFi in HGG, a key limitation of BRAFi is the transient nature of their efficacy, with tumor recurrence developing over time. This phenomenon has been well-documented in cancers such as melanoma, where reactivation of the MAPK pathway or activation of alternative survival pathways drives tumor regrowth despite continued BRAFi treatment. Similarly, this effect has been reported in HGGs with the acquisition of mutations in the *RAS*/*MAPK* pathway and histologic transformation to gliosarcoma^48^. Nonetheless, the promising initial response observed in some patients with HGG underscores the therapeutic potential of BRAFi.

More recently, *IDH1/2* mutations, present in up to 80% of low-grade gliomas (LGGs) and associated with favorable prognoses, have been investigated as therapeutic targets in gliomas. The phase III INDIGO trial (NCT04164901) demonstrated that vorasidenib, an IDH1/2 inhibitor, significantly improved median PFS (27.7 months versus 11.1 months with placebo) in patients with *IDH1*- or *IDH2*-mutant LGGs. Following these results, vorasidenib received FDA approval in 2024 for the treatment of *IDH* mutant LGGs. While these results are promising for LGGs, the role of IDH inhibitors in HGG remains uncertain with further trials necessary to define their role^49^.

It remains clear that genomics-driven strategies have failed to deliver the anticipated clinical success in HGGs. Nevertheless, precision oncology remains a promising strategy; however, realizing its full potential requires acknowledgement of its core principles and context-specific limitations. Ultimately, therapeutic efficacy depends not on a treatment’s intrinsic potency but on how well it aligns with the biological landscape it is applied to—a critical consideration that must guide future therapeutic development.

### Factors limiting the success of targeted therapies

Precision oncology was founded on the premise of identifying genetic or molecular changes that distinguish tumors from normal tissues, thereby enabling the selection of efficient treatment specific to the tumor. Fundamentally, the success of precision oncology depends on three key factors: identifying targetable variants, developing drugs that can effectively target these variants and ensuring the effective delivery of these targeted therapies. Challenges at any of these stages can hinder the success of targeted treatments. We discuss these factors in the context of HGG.

#### Identification of targetable variants

Large-scale cancer genomics initiatives, such as TCGA, International Cancer Genome Consortium (ICGC), and Genomics Evidence Neoplasia Information Exchange (GENIE) have facilitated the analysis of thousands of tumor genomes to identify targetable variants^50^. However, the identification of clinically actionable variants in HGG remains highly challenging. A major challenge in this process is the differentiation between true ‘driver mutations’, which promote cancer, from a vast majority of ‘passenger’ mutations, which accumulate without causal relevance to oncogenesis. This is further complicated by the fact that certain passenger mutations, although seemingly noncontributory in isolation, may act as ‘conditional’ driver mutations in the presence of other mutations^51–53^. Furthermore, current computational algorithms, which are principally guided by mutational frequencies, may overlook low-penetrance genetic events, thereby missing rare but potentially actionable drivers^54^. More importantly, even after identifying potential targets, our understanding of these variants often remains limited as the discovery of new variants outpaces our ability to classify them effectively^54^. Together, these challenges underscore the difficulty of reliably identifying targetable genomic variants in HGG.

Consequently, alternative approaches such as transcriptomic profiling have been pursued to better capture the dynamic nature of tumor biology. In HGGs, a landmark study by Verhaak et al. introduced gene expression-based classification of HGG into classical, mesenchymal and proneural subtypes^55^. Building on this, Neftel et al. demonstrated the existence of four cellular states—NPC-like (neural progenitor like), OPC-like (oligodendrocyte progenitor like), AC-like (Astrocyte like) and MES-like (mesenchymal like)—while highlighting the dynamic plasticity between states and the potential for a single cell to generate all four states^56^. Remarkably, a single tumor often contains a mixture of these states, with 60% of gliomas presenting with two or three different subtypes within the same tumor mass. Glioma stem-cells (GSCs) add further complexity, representing the apex cell population in HGG, underpinning phenotypic diversity, therapeutic resistance and recurrence^57^. Through self-renewal and multipotency, they regenerate diverse cellular lineages following treatment, while evading cytotoxic stress via enhanced DNA damage repair, metabolic adaptation, quiescence and activation of signaling networks^56,58^. The cellular diversity of GSCs, spanning distinct molecular subtypes and existing along gradients of transcriptional states with unique vulnerabilities, further exemplifies the difficulty of defining a single targetable vulnerability in HGG^59^.

#### Ability to target oncogenic drivers

Second, there remains a discordance in our ability to translate identified genetic or molecular targets into clinically relevant drugs. Approximately 60% of small-molecule drug discovery projects fail owing to identified targets considered ‘undruggable’. Prominent examples include RAS, MYC and fusion transcription factors, which are challenging to target owing to extensive protein–protein interaction interfaces and the absence of deep protein-binding sites. Loss-of-function mutations are particularly challenging to treat therapeutically, with frequently affected genes in HGG, such as *NF1*, *RB1*, *TP53*, *PTEN* and *ATRX*, remaining largely uncharted territory^60^.

In addition, delivering therapeutic agents to the brain is notoriously challenging owing to the combined effects of the blood–brain barrier (BBB) and the brain–tumor barrier. The BBB, a neurovascular construct regulating the passage of substances into healthy brain tissue, limits the penetration of over 98% of small drug molecules, with those that do cross often being expelled by efflux pumps such as P-glycoprotein. The brain–tumor barrier, driven by the secretion of factors such as vascular endothelial growth factor and hepatocyte growth factor, forms an abnormal vascular structure with heterogeneous permeability, creating hypoxic regions that impede drug delivery and promote resistance. These stringent barriers have contributed to the high failure rate of drug candidates during early development stages, with FDA approval rates for CNS drugs notably lower than those for non-CNS drugs^61^. Understanding this barrier, recent approaches have been developed to overcome the BBB and enhance the bioavailability and efficacy of therapeutic agents, including nanoparticles, hyperosmolar therapy, convection-enhanced delivery and magnetic resonance-guided focused ultrasound^62^.

#### Clinical efficacy of targets

Finally, it is imperative to note that only a small subset of precision oncology strategies have resulted in meaningful changes to cancer management, with the clinical impact of targeted therapy varying considerably across cancer types^63^.

Basket trials, designed to evaluate the efficacy of targeted therapies across multiple cancer types sharing the same actionable target, have become a widely employed strategy to evaluate the effectiveness of molecular-targeted therapies for oncogene-defined subsets of cancers. This approach offers the advantage of simultaneously evaluating therapies for multiple tumor types, including rare cancers, potentially accelerating the drug development process. However, clinical trial outcomes revealed significant limitations in patient–treatment matching and drug accessibility. The phase II SHIVA trial (NCT01771458) found that although 30–50% of patients harbored only driver mutations, only 26% received matched treatments^64^. Similarly, in the phase II NCI-MATCH trial (NCT02465060), 38% of patients were matched to targeted therapies, but only 18% received treatment^65^. A pooled analysis of basket trials involving 1100 patients across 33 different cancers found that targeted therapy guided by biomarkers associated with driver mutations achieved only a 25% response rate, highlighting the limitations of static biomarker-driven approaches^63^. Beyond these low matching rates, none of the trials demonstrated a significant improvement in PFS^63^. Consistent with these findings, a recent large-scale real-world meta-analysis reported that genomic profiling-guided therapy was delivered to 15.6% of patients with metastatic solid tumors, achieving an objective response rate of 23.9% and modest gains in PFS and OS, although the overall proportion of patients deriving benefit remained limited^66^.

These findings emphasize a central challenge in the practical application of precision oncology: even when genomic profiling successfully identifies actionable mutations, the clinical utility of this information remains heavily constrained by the availability of effective agents and the low proportion of patients who ultimately receive matched therapy. While continued innovation in the development of pharmacological targets is crucial, more can be done to optimize the use of existing agents that may work beyond genomically matched indications, allowing us to maximize the potential of current therapies.

Therapeutic responses are shaped by the confluence of intricate tumor–microenvironment interactions, epigenetic changes, phenotypic plasticity and factors far exceeding our current understanding or measurement capabilities^67^. Biomarker-driven strategies simplify these complexities by reducing gene and molecular interactions to binary terms—such as the presence or absence of a specific mutation—and measurable parameters focused solely on targets under investigation. These approaches assume that shortlisted parameters alone are sufficient to predict clinical outcomes, disregarding the broader biological influences that govern treatment response^68^. In reality, gene products rarely function in isolation, as discussed previously, but operate within highly interconnected networks where parallel systems can compensate for missing components^39^. Correspondingly, precision oncology has advanced toward integrative multiomics, combining genomic, transcriptomic, proteomic, metabolomic and epigenomic data, offering a panoramic view of tumor biology^69^. Recent integrative multiomics studies on HGG tumors have comprehensively characterized the disease, stratifying patients into distinct molecular subtypes, each with its own unique predicted susceptibilities^70–73^. While these approaches have deepened our understanding of the disease, current strategies are largely retrospective or predictive in nature, and prospective evidence demonstrating that multiomics data can directly inform clinical decision-making remains limited. Furthermore, the generation of vast, multidimensional datasets poses substantial translational challenges, particularly in analyzing such complex information in a clinically relevant manner that directly captures how these alterations converge to determine therapy response and can be readily leveraged by clinicians^69^.

Collectively, these challenges underscore the need for strategies that bridge the complex and dynamic molecular networks with functional therapeutic insights. FPO has emerged as a promising strategy to address this gap by directly linking functional responses of biological systems with actionable therapeutic implications, evaluating tumor cell behavior in its entirety rather than focusing on discrete molecular alterations^39^.

## FPO: the bridge between genotype and phenotype

FPO represents a transformative strategy that carries the potential to bridge the gap between molecular characterization and actionable therapeutic insights. Functional approaches that involve dynamic perturbations of tumor cells and the measurement of their responses capture the complexities and functional dependencies of cancer cells^74^. By harnessing the potential of existing drug libraries, FPO broadens the scope of treatment options available to patients, extending beyond molecular alterations and standard protocols. Prioritizing functional outputs over purely mechanistic understanding, FPO aligns with the need for clinically translatable strategies capable of addressing the heterogeneity and adaptability of malignancies, an approach that has shown promise in both pediatric and adult cancers^75^. This is especially noteworthy in the context of hard-to-treat, highly heterogeneous malignancies such as gliomas and sarcomas, and rare cancers, where limited molecular insights and a scarcity of effective treatment options pose considerable challenges to clinical management^76^. While FPO may be a relatively recent addition to the precision oncology landscape, its conceptual roots lie in foundational pharmacological practices: selecting therapeutic targets based on cellular responses, a principle through which virtually every chemotherapy agent used in oncology was derived from. This same principle is now applied at an individual patient level, allowing for personalized treatment strategies tailored to the specific functional characteristics of each patient’s cancer.

**Fig. 2:**
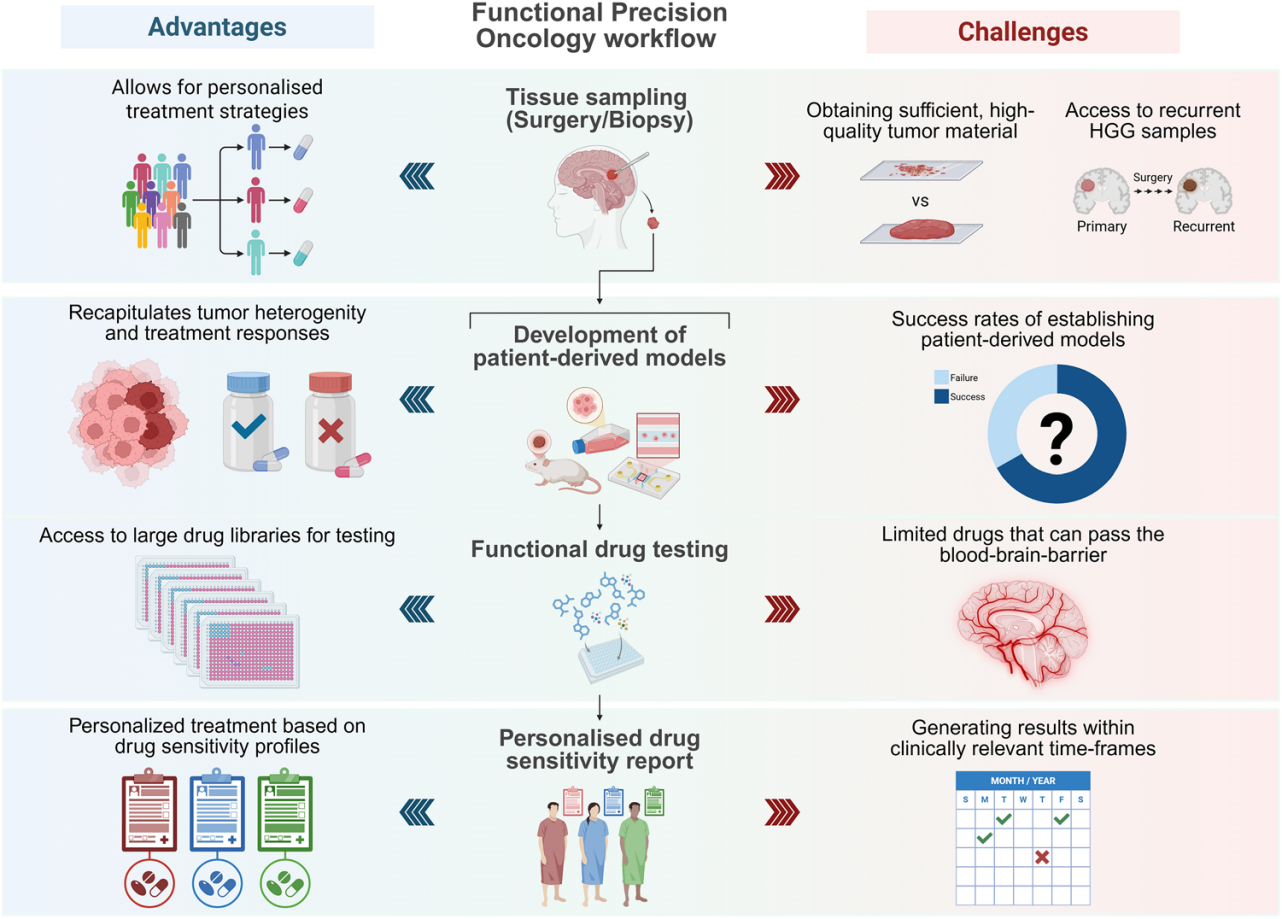
Workflow, advantages and challenges of FPO approaches in HGG. Patient-derived HGG samples can be obtained from primary or recurrent tumors through patient biopsies or resections. Tumor cells are isolated and processed to generate patient-derived tumor models, including organoids, tumorspheres or xenografts. These models undergo high-throughput drug screening to identify effective, personalized drug combinations, facilitating the development of individualized therapeutic strategies for patients with HGG.

FPO workflows commence with the collection of patient-derived samples, such as tumor tissue or blood, which are utilized to generate patient-derived models, including organoids, explants or spheroids. These models are then employed for drug screening and functional assays to inform personalized therapeutic strategies. This approach may be used to predict patient responses to SOC therapies, enabling improved patient stratification, while also expanding to a broader drug repository to facilitate the repurposing of chemotherapy and targeted agents (Fig. 2). A central assumption underpinning the success of this strategy is the ability of these models to faithfully retain the molecular and phenotypic fidelity of their parental tumor, which is crucial for accurate clinical extrapolation. Patient-derived xenograft (PDX) models have been shown to faithfully recapitulate tumor features with high fidelity, accurately reflecting the biological characteristics and therapeutic responses observed in the original patient tumors^77^. In PDX models of HGGs, in addition to genomic, morphological and pathological similarities, patient-specific responses to TMZ and radiotherapy as part of the SOC treatment were successfully replicated^78^. Similarly, patient-derived organoids have demonstrated strong genotypic and phenotypic concordance with their parental tumors, including histological features, cellular diversity, gene expression and mutational profiles of corresponding parental tumors^79^. A growing area of research has focused on enriching these models for GSC populations, given their rarity and tendency to be missed by bulk genomic or transcriptomic analyses^80^. Enrichment of GSC populations has been successfully achieved through the establishment of patient-derived gliomaspheres, which serve as in vitro models that selectively expand stem-like cell populations from tumor samples for downstream functional assays^81,82^. Such enrichment enables direct interrogation of the drug sensitivities and adaptive resistance mechanisms of the very cells that drive recurrence, thereby guiding the development of more effective, patient-tailored therapies. Emerging platforms, such as microfluidics and engineered microenvironments aim to further recapitulate molecular and mechanical cues in human tissues^83^. Although the overarching concept of the various patient-derived models are the same, model-specific drawbacks and challenges that are cancer specific exist that need to be considered when selecting the appropriate model^84^.

While FPO approaches are still emerging, they have demonstrated notable success across multiple cancer types in both pediatric and adult populations (Tables 1 and 2). In pediatric patients with relapsed or refractory malignancies, a prospective observational study (NCT03860376) demonstrated that FPO-derived treatments significantly improved PFS by more than 1.3-fold in 83% of pediatric cases compared to prior treatments^85^. Encouraged by these findings, ongoing trials are expanding this approach to larger cohorts of children (NCT05857969) and adults (NCT06024603)^85^. In adults, EXALT-1, a phase IIb trial (NCT03096821), was the first precision oncology trial to use functional assays to guide the identification of personalized therapies^86^. At a median follow-up of 23.9 months, 54% of patients demonstrated a more than 1.3-fold improvement in PFS compared to prior therapies, with 40% achieving exceptional responses^86^. Notably, 23% of patients remained progression-free at 12 months after FPO-guided therapy, significantly outperforming the 5% progression-free rate observed with previous treatments^86^. This work forms the basis for the recently initiated prospective randomized trial EXALT-2 (NCT04470947)^87^. Since then, several other trials have been initiated across both hematological malignancies and solid tumors, albeit considerably fewer in the latter^75,88,89^.

**Table 1:** Summary for FPO trials internationally excluding indications for HGGs. Trial details retrieved from ClinicalTrials.gov, accessed April 2025^75,88,89^.

Study title	Type of cancer	Type of patients	Estimated enrollment	NCT number	Study location	Study type	Status	Start date	Expected date of completion	References (where available)
Targeted Therapy Selection Based on Tumor Tissue Kinase Activity Profiles for Patients with Advanced Solid Malignancies, an Exploratory Study (TSAP)	Advanced (unresectable and/or metastatic) solid malignancy for whom no standard treatment is available	Adults	45	NCT01190241	TN, USA	Interventional	Terminated	01 August 2010	01 December 2027	^108^
Personalized Patient Derived Xenograft (pPDX) Modelling to Test Drug Response in Matching Host	Colorectal, breast, and ovarian neoplasms	Adults	120	NCT02732860	Ontario, Canada	Observational	Recruiting	01 December 2015	04 January 2026	Nil
Beat AML Core Study	Acute myeloid leukemia (AML)	Adults (>18 years)	22	NCT02927106	FL, USA	Observational	Completed	15 February 2017	29 July 2020	Nil
Study of the Therapeutic Response and Survival of Patients with Metastatic Colorectal Cancer (stage IV) and Treated According to the Guidelines of a Chemosensitivity Test, Oncogramme (ONCOGRAM) (ONCOGRAM)	Metastatic colorectal cancer	Adults (>18 years)	256	NCT03133273	Limoges, France	Interventional	Completed	24 July 2017	03 April 2024	^109^
PRecISion Medicine for Children With Cancer (PRISM)	High-risk childhood cancers with expected survival < 30%	Up to 21 years old	550	NCT03336931	Sydney, Australia	Observational	Active, not recruiting	05 September 2017	01 December 2023	^110^
Personalized Precision Diagnosis and Treatment of Pancreatic Cancer (PPDTPC)	Pancreatic cancer	Adults (20 years to 75 years)	100	NCT04373928	Shanghai, China	Interventional	Recruiting	10 November 2017	31 December 2024	Nil
High Throughput Drug Sensitivity and Genomics Data in Developing Individualized Treatment in Patients with Relapsed or Refractory Multiple Myeloma or Plasma Cell Leukemia	Plasma cell leukemia/recurrent and relapsed multiple myeloma	Adults (>18 years)	40	NCT03389347	Seattle, WA, USA	Interventional	Active, not recruiting	14 February 2018	19 December 2026	^111^
Patient-Derived Xenografts in Personalizing Treatment for Patients with Relapsed/ Refractory Mantle Cell Lymphoma	Relapsed/ Refractory Mantle Cell Lymphoma	Adults (>18 years)	1	NCT03219047	TX, USA	Interventional	Completed	20 December 2018	03 July 2023	Nil
Ex Vivo Drug Sensitivity Testing and Mutation Profiling	Recurrent or refractory cancer	Up to 21 years old	25	NCT03860376	FL, USA	Observational	Completed	21 February 2019	31 December 2022	^85^
The PIONEER Initiative: Precision Insights on *N*-of-1 Ex Vivo Effectiveness Research Based on Individual Tumor Ownership (Precision Oncology) (PIONEER)	Any cancer	1 Month to 99 years (child, adult, older adult)	1000	NCT03896958	GA, USA	Observational	Unknown status	21 March 2019	12 March 2024	Nil
Novel 3D Hematological Malignancy Organoid to Study Disease Biology and Chemosensitivity (Organoid)	Hematologic malignancy	Adults (>18 years)	70	NCT03890614	NC, USA	Observational	Recruiting	16 May 2019	01 February 2026	Nil
Q-GAIN (Using Qpop to Predict Treatment for Gastrointestinal caNcer)	Gastrointestinal cancers	Adults	100	NCT04611035	Singapore	Observational	Unknown status	20 January 2020	01 January 2023	Nil
Serial Measurements of Molecular and Architectural Responses to Therapy (SMMART) PRIME Trial	metastatic solid tumor or hematological malignancy	Adults (>18 years)	2	NCT03878524	OR, USA	Interventional	Terminated	01 April 2020	10 December 2020	Nil
Comprehensive Genomic Profiling and Next Generation Functional Drug Screening for Patients with Aggressive Hematological Malignancies	Advanced and refractory lymphoma/refractory leukemia/refractory AML/refractory T cell lymphoma	Adults	150	NCT04470947	Vienna, Austria	Observational	Recruiting	10 June 2020	31 March 2026	^112^
Feasibility Study of Multi-Platform Profiling of Resected Biliary Tract Cancer	Biliary Tract Cancer	Adults (>18 years)	14	NCT04561453	Washington, USA	Observational	Active, not recruiting	08 July 2020	01 July 2026	^113,114^
Using QPOP to Predict Treatment for Sarcomas and Melanomas	Sarcoma, melanoma	Adults	100	NCT04986748	Singapore	Observational	Recruiting	08 September 2020	31 December 2028	^115^
Hyper-Personalized Medicine Using Patient Derived Xenografts (PDXovo) for Metastatic Solid Tumors (PDXovo)	Metastatic solid tumors	Adults (>18 years)	50	NCT04602702	Ontario, Canada	Observational	Unknown status	01 December 2020	31 December 2022	Nil
Guided Treatment Based on Mini-PDX in Metastatic Triple Negative Breast Cancer	Triple Negative Breast Cancer	Adults (>18 years)	100	NCT04745975	Shanghai, China	Interventional	Unknown status	01 February 2021	01 January 2023	Nil
FPO for Metastatic Breast Cancer	HER2-negative breast cancer	Adults	15	NCT04450706	UT, USA	Interventional	Completed	16 February 2021	05 September 2024	Nil
3D Bioprinted Models for Predicting Chemotherapy Response in Colorectal Cancer with/without Liver Metastases	Colorectal cancer	Adults (>18 years)	120	NCT04755907	Beijing, China	Observational	Unknown status	01 March 2021	31 December 2023	^116^
High Throughput Screening Device Based on 3D Nano-matrices and 3D Tumors with Functional Vascularization (TUMOVASC)	Non-small cell bronchopulmonary cancer (NSCLC)	Adults (>18 years)	100	NCT04826913	Strasbourg, France	Observational	Unknown status	01 April 2021	01 April 2024	Nil
Organoid-Guided Chemotherapy for Advanced Pancreatic Cancer	Pancreatic cancer	Adults (>18 years)	100	NCT04931381	Shanghai, China	Interventional	Recruiting	01 June 2021	31 May 2025	Nil
Organoid-Guided Adjuvant Chemotherapy for Pancreatic Cancer	Pancreatic cancer	Adults (>18 years)	200	NCT04931394	Shanghai, China	Interventional	Recruiting	01 June /2021	31 May 2025	Nil
Patient-Tailored Hyperthermic Intraperitoneal Chemotherapy (HIPEC) for Colorectal Peritoneal Metastases (OrganoHIPEC)	Peritoneal metastases from colorectal cancer	Adults	24	NCT06057298	Milan, Italy	Interventional	Recruiting	15 June 2021	14 June 2025	^117^
Quadratic Phenotypic Optimization Platform (QPOP) utilization to Enhance Selection of Patient Therapy Through Patient Derived Organoids in Breast Cancer	Breast cancer	Adults	26	NCT05177432	Singapore	Interventional	Recruiting	06 December 2021	31 December 2025	Nil
Systemic Neoadjuvant and Adjuvant Control by Precision Medicine in Rectal Cancer (SYNCOPE)	Rectal cancer	Adults (>18 years)	93	NCT04842006	Helsinki, Finland	Interventional	Recruiting	20 December 2021	31 December 2031	Nil
Drug Response Profiling (DRP) Registry Zurich for Hematological Malignancies (DRP_ZH)	Primary, relapsed or refractory hematological malignancy (leukemia, myeloma, or lymphoma)	Children and adults (≤40 years)	1,000	NCT06550102	Zurich, Switzerland	Observational	Recruiting	04 January 2022	31 December 2031	Nil
Prospective Multicenter Study Evaluating Feasibility and Efficacy of Tumor Organoid-based Precision Medicine in Patients with Advanced Refractory Cancers (ORGANOTREAT)	Advanced solid tumors	Adults (>18 years)	61	NCT05267912	Paris, France	Interventional	Active, not recruiting	19 January 2022	18 January 2028	^118^
Tailoring treatment in colorectal cancer	Colorectal neoplasms	Adults	40	NCT05401318	Viken, Norway	Observational	Recruiting	28 March 2022	01 January 2027	Nil
The Clinical Efficacy of Drug Sensitive Neoadjuvant Chemotherapy Based on Organoid versus Traditional Neoadjuvant Chemotherapy in Advanced Gastric Cancer	Gastric cancer	Adults ( > 18 years)	54	NCT05351398	Shanghai, China	Observational	Unknown status	01 April 2022	01 December 2023	Nil
The Culture of Ovarian Cancer Organoids and Drug Screening	Ovarian cancer	Adults ( > 18 years)	30	NCT04768270	Chongqing, China	Observational	Recruiting	12 April 2022	31 December 2024	^119^
Optimizing and Personalizing Azacitidine Combination Therapy for Treating Solid Tumors QPOP and CURATE.AI	Breast or Gastric cancer	Adults	10	NCT05381038	Singapore	Interventional	Not yet recruiting	01 June 2022	04 April 2027	Nil
Patient-Derived Organoid (PDO) Guided Versus Conventional Therapy for Advanced Inoperable Abdominal Tumors	Abdominal tumors	Adults ( > 18 years)	0	NCT05378048	Hong Kong	Interventional	Withdrawn	04 July 2022	03 July 2025	Nil
Study to Investigate Outcome of Individualized Treatment in Patients with Metastatic Colorectal Cancer (EVIDENT)	Metastatic colorectal cancer	Adults	40	NCT05725200	Oslo, Norway	Interventional	Recruiting	27 September 2022	31 December 2040	Nil
The Clinical Efficacy of Drug Sensitive Neoadjuvant Chemotherapy Based on Organoid versus Traditional Neoadjuvant Chemotherapy in Advanced Rectal Cancer	Rectal cancer	Adults ( > 18 years)	192	NCT05352165	Shanghai, China	Interventional	Not yet recruiting	01 January 2023	31 December 2025	Nil
FPO to Predict, Prevent, and Treat Early Metastatic Recurrence of TNBC	Recurrent breast cancer	Adults	80	NCT05464082	UT, USA	Interventional	Recruiting	06 January 2023	30 September 2027	^120^
Guiding Instillation in Non-Muscle-Invasive Bladder Cancer Based on Drug Screens in PDOs	Bladder cancer/nonmuscle invasive	Adults	34	NCT05024734	Biel, Switzerland	Interventional	Recruiting	21 February 2023	31 October 2027	^121^
Ex Vivo Drug Sensitivity Testing and Multi-omics Profiling	Recurrent childhood cancer (all types)	Children	65	NCT05857969	FL, USA	Observational	Recruiting	22 February 2023	31 December 2028	^122^
Individualized Treatments in Adults with Relapsed/refractory Cancers	Refractory/relapsed cancer (all types)	Adults	36	NCT06024603	FL, USA	Interventional	Recruiting	20 November 2023	01 November 2025	Nil
Pharmacoscopy-Guided Clinical Standard-of-Care in R/r AML (RAPID-01)	Relapsed/refractory AML	Adults	88	NCT06138990	Zurich, Switzerland	Interventional	Recruiting	02 September 2024	30 June 2026	Nil

**Table 2:** Summary of FPO trials in HGGs internationally; trial details retrieved from ClinicalTrials.gov, accessed April 2025^75,88,89^.

Study title	Type of cancer	Type of patients	Estimated enrollment	NCT number	Study location	Study type	Status	Start date	Expected date of completion	References (where available)
Evaluation of ex vivo drug combination optimization platform in recurrent high grade astrocytic glioma	Glioma, astrocytic	Adults	10	NCT05532397	Singapore	Interventional	Recruiting	17 February 2023	01 December 2025	^92^
Personalized targeted glioblastoma therapies by ex vivo drug screening (ATTRACT)	Glioblastoma	Adults	240	NCT06512311	Vienna, Austria	Interventional	Recruiting	10 July 2024	31 December 2031	^93,94^
PTCs-based Precision Treatment Strategy on Recurrent HGGs	Recurrent HGGs	Adults	30	NCT05473923	Beijing, China	Interventional	Active, not recruiting	12 August 2022	30 June 2025	Nil
Pilot trial for treatment of recurrent glioblastoma	Recurrent IDH wild-type glioblastoma	Adults	10	NCT05432518	Alberta, Canada	Interventional	Recruiting	27 June 2023	01 December 2027	Nil
Ex vivo determined cancer therapy (EVIDENT)	Bladder cancer/kidney cancer/ melanoma/ sarcoma/ glioblastoma/ head and neck cancer	Children (>16 years) and adults	600	NCT05231655	Sheffield, UK	Observational	Recruiting	07 July 2021	01 January 2027	^123^

In HGG, four ongoing FPO trials are employing ex vivo approaches in guiding the management of primary and recurrent tumors (Table 2). Among them, the phase II EVIDENT trial (NCT05231655), part of a pan-cancer study on solid tumors, and the phase II 3D-PREDICT trial (NCT03561207) employ functional testing in treatment-naive HGG to predict response to SOC treatment. Preliminary results from the 3D-PREDICT study demonstrated the ability to identify TMZ responders irrespective of *MGMT* methylation status, with the identification of patients who responded to TMZ despite being *MGMT* unmethylated. Test-predicted responders had a median OS postsurgery of 11.6 months compared to 5.9 months for test-predicted nonresponders. With functional data provided within 7–10 days of tissue receipt, patients with HGG whose tumors are predicted not to respond favorably to TMZ could be preferentially directed to clinical trials or managed in ways that might offer greater clinical benefit^90^. Currently, *MGMT* promoter methylation status is the primary metric used to predict responses to TMZ clinically. However, its limitations are well-documented, as both methylated patients who respond poorly to therapy, and unmethylated patients who respond favorably have been observed in numerous studies. Consequently, while *MGMT* methylation status may serve as a predictor of response, its impact on clinical decision-making regarding TMZ administration remains limited^91^. Its role is also limited owing to the lack of approved alternative cytotoxic therapies in the first-line setting. These findings highlight the importance of complementing static biomarkers such as *MGMT* methylation status with functional drug studies to improve the selection of therapeutic targets for patients. Other ongoing trials include the phase Ia/IIb trial (NCT05532397) for recurrent HGG, which utilizes the quadratic phenotypic optimization platform (QPOP), a computational analytic platform designed to identify top ranking drug combinations and the phase II ATTRACT trial (NCT06512311), which similarly employs ex vivo drug sensitivity testing to personalize treatment strategies for patients^92–94^.

In addition to the identification of personalized drug targets, functional drug screens have played a pivotal role in accelerating therapeutic discovery. A notable example is the identification of panobinostat as a potential therapy for pediatric diffuse midline gliomas following a drug screen of 83 compounds. These findings have progressed to several phase I clinical trials (NCT02717455, NCT03566199 and NCT03632317)^95^. Similarly, in adult HGGs, functional screens across 132 neuroactive drugs revealed that the antidepressant vortioxetine synergizes with current SOC chemotherapies in vivo^96^. Furthermore, functional screens also provide valuable mechanistic insights, elucidating the mechanisms of action of existing approved drugs, thereby optimizing their clinical use. For instance, the lack of efficacy of BRAFi in *BRAF*^*V600E*^-mutated colorectal cancers, despite their success in melanoma with the same mutation, was clarified through functional studies, which identified feedback activation of *EGFR* in colorectal tumors^97^. Together, these examples highlight the potential of FPO both in driving therapeutic innovation and in refining the clinical application of existing agents through deeper mechanistic understanding of tumor biology.

Delivering drug sensitivity profiles within clinically relevant timelines remains a crucial hurdle, requiring efficient clinic-to-bench workflows and rapid generation of results. Achieving this demands close collaboration between neurosurgeons, oncologists, pathologists and scientists. Ideally, laboratories conducting FPO analysis should be located near major hospitals performing brain tumor resections to ensure timely access to high-quality tumor samples. A major obstacle in HGG is the acquisition of sufficient, high-quality tumor material for analysis, which often requires invasive surgical resections or biopsies. This limitation is exacerbated in deep-seated or recurrent tumors, where less than 10% of recurrent HGGs are eligible for resection. In addition, factors such as tumor necrosis and preoperative steroid use frequently impede successful ex vivo expansion of tumor cells, with these challenges particularly pronounced in the context of recurrent tumors^84^. Despite these obstacles, studies by Jacob et al. have demonstrated the feasibility of generating and biobanking patient-derived HGG organoids, achieving an overall success rate of 91.4%^79^. However, success rates vary depending on the tumor subtype, with lower success rates observed for *IDH1*-mutant tumors (66.7%) and recurrent tumors^79^. Most recently, a 2025 study indicated a 66.5% success rate for establishing patient-derived HGG organoids^98^, while other studies have reported success rates of 80–90% in HGG PDX models^78^.

Another key challenge of current models is their inability to replicate key aspects of the tumor microenvironment necessary for evaluating specific therapies. Therapeutic agents such as bevacizumab, which target angiogenesis cannot be effectively tested in many current models owing to their inability to replicate the vascular architecture required for assessing such treatments. Similarly, immunotherapies require advanced 3D culture systems capable of replicating immune–tumor interactions, as traditional models, including 2D systems and basic tumorspheres models lack the complexity needed to evaluate these therapies effectively. The development of advanced platforms, including microfluidic systems, cerebral organoids and humanized PDX models, has broadened the range of therapies^99^. In HGG, preclinical studies have demonstrated the feasibility of co-culturing organoids with chimeric antigen receptor T cells for therapeutic testing^84^. Notably, ongoing evaluations in six patients involve real-time parallel assays using the same therapeutics administered to patients, providing prospective insights into treatment responses^100,101^.

Although FPO theoretically enables the screening of an extensive range of therapeutic compounds, its practical application remains constrained by challenges such as limited tumor sample availability and the labor-intensive nature of comprehensive drug screening. In addition, with combinatorial strategies gaining prominence in oncology, the identification of effective drug combinations has become a priority in translational research. However, large-scale studies have shown that true synergies are uncommon and challenging to detect^102,103^. Development of biology-guided screening approaches that incorporate molecular context and mechanistic insights have shown to further improve the likelihood of uncovering clinically meaningful combinations from high-throughput screening strategies^104^. An emerging opportunity is to integrate these approaches with AI-based models, such as DeepSynergy and COSTAR, which could enable the assimilation of complex datasets and improve the prediction of synergistic drug pairs^96,105^. Such integration would build on the reciprocal and interdependent nature of genomics and FPO, with each providing critical insights that enhance the effectiveness of the other.

**Fig. 3:**
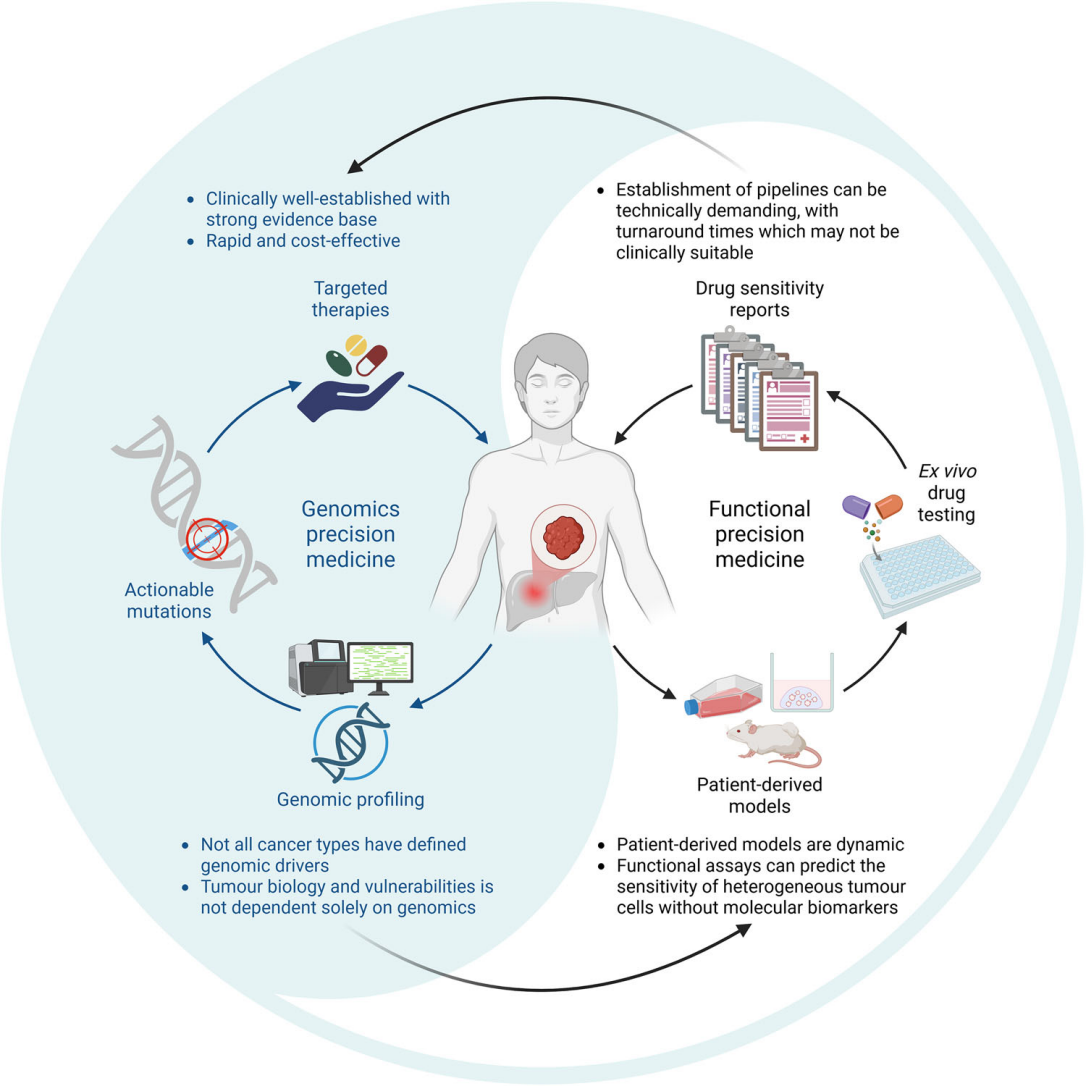
Complementary strengths of genomic and functional precision medicine. Genomic and functional approaches provide distinct but complementary insights for guiding treatment. Genomics enables rapid, evidence-based identification of actionable mutations, but may overlook nongenomic vulnerabilities. Functional testing, though more technically demanding, captures tumor dynamics and drug responses beyond what is detectable through molecular profiling.

While FPO holds great potential to transform HGG management through personalized, biology-driven therapeutic strategies, it remains in its early stages and several challenges must be addressed before it can be widely adopted in clinical practice. Current evidence is largely derived from single-arm trials in highly selected patient populations, requiring cautious interpretation. The lack of randomized, prospective trials means that the true clinical benefit of FPO has yet to be definitively established, with future efforts focused on validating FPO within structured clinical trial frameworks. Furthermore, the utility of ex vivo drug platforms must be approached with nuance as they may demonstrate responses to targeted therapies even in the absence of the corresponding molecular target, such as BRAFi showing activity in tumors lacking *BRAF* mutations^106^. These discrepancies highlight the complexity of tumor biology, suggesting the need for a more comprehensive approach that integrates ex vivo results with genomic, transcriptomic and proteomic data, where cross-validation may enhance the accuracy of tumor vulnerability assessments and improve treatment selection (Fig. 3).

It is imperative to acknowledge that while FPO approaches, based on *N*-of-1 trial designs offer many advantages, they are not suitable or necessary for all cancers^107^. These trials are resource intensive, requiring substantial time, effort and financial investment. Their personalized nature complicates scalability, making integration into routine clinical practice challenging. Furthermore, regulatory frameworks and evidence-based guidelines, designed for population-level efficacy, provide limited support and few incentives for such individualized approaches. The selection of treatment strategies and trial design should be guided by the unique characteristics of the cancer in question, ensuring alignment with its biological complexity and therapeutic challenges. For cancers with well-defined SOCs that are effective for the majority of patients, population-based trials may remain the most practical approach. By contrast, rare, refractory or highly heterogeneous cancers that lack an established SOC or perform poorly under existing treatments may benefit from *N*-of-1 trials^107^. In this context, personalized treatment strategies enabled by FPO-based *N*-of-1 trials present a promising alternative for HGGs, offering hope where conventional approaches have consistently fallen short.

## Conclusion and future perspectives

In summary, despite two decades of effort, precision oncology in HGG has fallen short of delivering transformative clinical outcomes. FPO holds promise as a complementary approach, moving beyond molecular profiling to incorporate patient-specific models that capture the unique biology of each tumor. Advancing the scalability of patient-derived models and integrating them with high-throughput drug screening and molecular profiling are crucial for positioning FPO as a foundational approach for both therapeutic development and personalized patient treatment. A critical challenge remains in translating these insights into clinical workflows. Achieving this will require coordinated efforts among multidisciplinary teams, including oncologists, pathologists, bioinformaticians and laboratory scientists, to streamline sample processing, ensure robust data interpretation and deliver individualized treatment strategies.

## Availability of data and materials

Data for this Review were identified through searches of PubMed, MEDLINE, ClinicalTrials.gov and references from relevant articles using the terms ‘functional precision oncology’, ‘high-grade glioma’, ‘patient-derived models’, ‘drug screening’, ‘clinical trials’ and ‘genomics’. Abstracts and meeting reports were included only when directly related to published work. Only articles published in English between 1975 and April 2025 were considered.
